# Generating regionalized neuronal cells from pluripotency, a step-by-step protocol

**DOI:** 10.3389/fncel.2012.00064

**Published:** 2013-01-03

**Authors:** Agnete Kirkeby, Jenny Nelander, Malin Parmar

**Affiliations:** Department of Experimental Medical Science and Lund Stem Cell Center, Lund UniversityLund, Sweden

**Keywords:** human embryonic stem cells, pluripotent stem cells, differentiation, protocol, neuronal subtypes, regionalization, GSK3, brain development

## Abstract

Human pluripotent stem cells possess the potential to generate cells for regenerative therapies in patients with neurodegenerative diseases, and constitute an excellent cell source for studying human neural development and disease modeling. Protocols for neural differentiation of human pluripotent stem cells have undergone significant progress during recent years, allowing for rapid and synchronized neural conversion. Differentiation procedures can further be combined with accurate and efficient positional patterning to yield regionalized neural progenitors and subtype-specific neurons corresponding to different parts of the developing human brain. Here, we present a step-by-step protocol for neuralization and regionalization of human pluripotent cells for transplantation studies or *in vitro* analysis.

## Introduction

Given that the human brain is far more complex than brains of rodents, chicks, or invertebrate model organisms, it is clear that knowledge of human brain development cannot be fully extrapolated only from animal models. Therefore, studies on human fetal tissue in combination with a reliable human *in vitro* modeling system are required. Due to very limited access to fetal tissue at different developmental time points, human cell sources such as pluripotent cells are necessary to allow for comprehensive, dynamic analysis of human neural development, and to generate sufficient amounts of cells for future regenerative therapies (Kirkeby and Parmar, 2012).

Based on previously published methods for neural differentiation (Zhang et al., 2001; Perrier et al., 2004; Nat et al., 2007; Chambers et al., 2009; Fasano et al., 2010), we have developed and refined a standardized protocol for rapid neural conversion of pluripotent cells using defined culture settings free of feeder cells, matrigel, knockout serum replacement (KSR), and manual picking steps (Kirkeby et al., 2012). By mimicking developmental cues important for neural tube patterning in mammals, the cells can be efficiently patterned to regionalized cultures of a high purity. The protocol presented here is based on rapid neural conversion through dual SMAD inhibition (Chambers et al., 2009) and regionalization of the cells in this protocol constitutes a flexible system controlled by dose-dependent chemical inhibition of glycogen synthase kinase 3 (GSK3) for rostro-caudal patterning and dose-dependent activation of sonic hedgehog (SHH) and bone morphogenetic protein (BMP) signaling for dorso-ventral patterning (Kirkeby et al., 2012).

## Differentiation procedure

**Figure 1:**
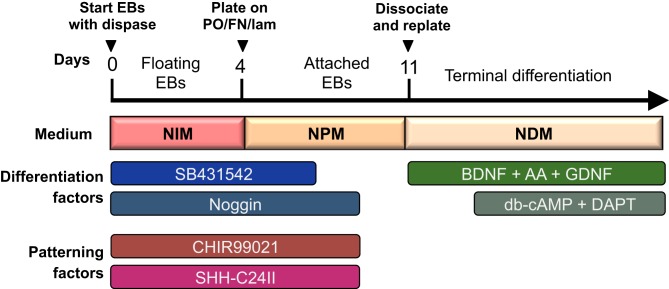
**Overview of differentiation protocol.** Cells are differentiated as EBs for the first 4 days, and then cultured as attached colonies on coated plates with subsequent dissociation and replating at day 11. Neuralisation and patterning factors are present between day 0–9 (only SB431542 is withdrawn at day 7), and terminal differentiation is initiated from day 14 and onward with db-cAMP and DAPT.

The protocol presented here will produce neural cells of a telencephalic fate in the absence of any patterning factors. To control rostro-caudal and dorso-ventral patterning, see separate section below. This protocol is preferably started on a Monday to avoid medium changes during the weekends (see overview of differentiation procedure in Figure 1). It is important to start the differentiation with healthy, pluripotent cells. All differentiated colonies should be removed from the culture before initiating differentiation.

**Optional**: If pluripotent cells are cultured on mouse embryonic fibroblasts (MEFs), colonies can be passaged onto matrigel in conditioned media or onto vitronectin in Essential 8 medium for 1 passage (3–7 days) before starting differentiation. This step will remove MEFs from the culture. Differentiation may also be initiated from colonies on MEFs, but if too many MEFs are present in the culture, this may result in attachment of embryoid bodies (EBs) to the bottom of the culture dish.

1. **Day 0**: Check that the colonies appear pluripotent by visual criteria, and if needed, remove differentiated colonies from the culture. Aspirate hESC media and wash cells once in PBS. Add minimal volume of EDTA (0.5 mM) to the cells (i.e., 0.5 ml for a 6-well) and incubate at 37°C for 5–8 min until colonies are easily washed off the dish with a pipette.
2. Gently pipette colonies off the dish and spin down in 10 ml culture medium at 200× g for 5 min.
  **NOTE**: When dissociating cells with EDTA, a washing step is not necessary. Here, cells are spun down to avoid dilution of the differentiation medium with EDTA solution. Alternatively, colonies can be enzymatically dissociated with dispase, but this requires additional washing steps and may result in a lower cell yield.
3. To start differentiation, re-suspend the cells in differentiation medium: NIM + Y-27632 (10 μM) + SB431542 (10 μM) + noggin (100 ng/ml). The volume of differentiation medium will depend on the amount of starting material. In general, the volume of differentiation medium should be equivalent to 1–1.5× the volume of growth medium used for the undifferentiated cells. That is, if starting with cells from 1 well of a 6-well plate, the differentiation volume should be 1.5–2.5 ml depending on the density of the cells. Plate cell suspension in non-treated cell culture plates for EB formation (i.e., 0.5 ml/well in a 24-well plate or 1 ml/well in a 12-well plate). Optional patterning factors can be added to the medium as needed (see section “Rostro-caudal and Dorso-ventral patterning of neural progenitors”).
  **NOTE**: ROCK inhibitor is added to the differentiation medium from day 0–2 to increase survival of EBs. If Fc-conjugated noggin is used, add 200 ng/ml noggin instead of 100 ng/ml. For some cell lines, 10 μM SB431542 may appear toxic, and the concentration should then be lowered to 2–5 μM. Start coating with poly-ornithine (PO) on this day.
4. **Day 2**: Transfer EB suspensions to a tube and spin down gently at 80× g for 5 min. Aspirate the medium and re-suspend the EBs in new NIM medium + SB431542 (10 μM) + noggin (100 ng/ml) + optional patterning factors using the same volume as on day 0. Return EBs to the wells they came from.
  **NOTE**: If necessary, wash wells with medium to retrieve any EBs that have attached to the well bottom. If big aggregates of EBs are present, these should be broken up with a pipette. If medium is very yellow by day 2, increase the volume of differentiation medium. Coat plates with fibronectin and laminin (FN/lam) on this day.
5. **Day 4**: Transfer EB suspensions to a tube and spin down gently at 80× g for 5 min. Resuspend EBs in NPM medium + SB431542 (10 μM) + noggin (100 ng/ml) + optional patterning factors. The volume of differentiation medium should now be 2× the previous volume. Transfer EBs to wells coated with PO/lam/FN (see section “Reagent Setup”) corresponding to a 1:2 split. If big aggregates of EBs are present, these should be broken up with a pipette before plating.
6. **Day 7**: Replace medium with NPM medium + noggin (100 ng/ml) + optional patterning factors. Coat with PO on this day.
7. **Day 9**: Replace medium with plain NPM medium without growth factors or patterning factors. Coat with FN/lam on this day.
8. **Day 11**: Replating: Wash cells with PBS, and add minimal volume of accutase to the wells. Leave cells in the incubator for 10 min or longer, until cells are easily suspended from the dish with a pipette. Dissociate cells with a pipette and spin down at 300× g for 5 min (before spinning, take an aliquot out for cell counting). Resuspend cells to a density of 5–15,000 cells/μl in NDM medium. Wash freshly coated plates with PBS and remove all liquid from the plates with a vacuum aspirator to achieve completely dry wells. Plate cells in droplets of 5–100 μl depending on future use. If many small wells are needed for immunocytochemistry, droplets of 5 μl can be plated in individual wells of 48-well plates to yield multiple replicates. If large amounts of cells are needed for transplantation or sorting, all cells can be plated in a single large droplet. Leave dishes in the incubator for 15–20 min to allow cells to attach to the well bottom. When attachment is verified in the microscope, add medium to the wells: NDM + BDNF (20 ng/ml) + GDNF (10 ng/ml) + AA (0.2 mM).
  **NOTE**: The cell density at this point is critical for the survival and proper differentiation of the cells, and optimal density should be adapted for each individual purpose. The cells need to be maintained at a very high density to avoid cell death and selective loss of neural subtypes. On the other hand, if the cell density is too high, neuronal maturation will be attenuated.
9. **Day 14**: Change medium to NDM + BDNF (20 ng/ml) + GDNF (10 ng/ml) + ascorbic acid (0.2 mM) + db-cAMP (500 μM) + DAPT (1 μM). Maintain the cells in this medium from now and onward to induce terminal neuronal maturation.

**NOTE**: Cells are at an optimal stage for transplantation between days 14–20 of differentiation. If cells are to be used for transplantation, avoid adding DAPT to the medium, as this will result in premature neuronal maturation causing increased cell death upon dissociation.

**Optional**: If cells are needed for long-term studies of mature neuronal phenotype, it may be necessary to replate cells again between days 16 and 20 to avoid too high density of the cultures. In that case, perform replating using the same procedure as on day 11. Replating at later timepoints should be avoided due to stress on mature neuronal cells. At late stages of differentiation, neurons may begin to detach from the plate. This can be attenuated by adding lam + FN to the cell culture medium.

### Rostro-caudal patterning of neural progenitors

Rostro-caudal patterning of the cells can be controlled by dose-dependent addition of the GSK3 inhibitor CHIR99021/CT99021. The compound should be added to the cells from day 0 to 9 of differentiation at an appropriate concentration for the purpose needed. Use 0.2–0.4 μM for diencephalic fates; 0.6–0.8 μ M for mesencephalic fates, 1–2 μM for anterior rhomencephalic fates and ≥4 μM for posterior rhomencephalic fates. Since different cell lines may show variations in the response to GSK3 inhibition, we recommend that each lab performs a dose-response curve of the CHIR99021 to determine the optimal concentration needed for generating the wanted cell type in their settings. Read section “Reagent Setup” for instructions on CHIR99021 preparation.

### Dorso-ventral patterning of neural progenitors

Ventralization of the cells can be obtained by adding Shh-C24II to the medium from day 0 to 9 of differentiation at any given concentration of CHIR99021. If no Shh is added to the culture, the cells will be enriched for alar plate fates. To enrich for basal plate, add 50–150 ng/ml, and to enrich for floor plate, add ≥200 ng/ml Shh-C24II. If more potent ventralization is wanted, purmorphamine (0.5 μM) can be added together with Shh from day 2 to 9. To achieve dorsalization of cultures toward roof plate fates, remove SB431542 and noggin from the cultures at day 4 to allow for BMP activation.

**NOTE**: Chemical compounds are generally used at lower concentrations in this protocol compared to protocols using knockout serum replacement (KSR) in the differentiation medium (Kriks et al., 2011). This may be due to a higher protein content and chemical buffering capacity in media containing KSR, necessitating higher concentrations of chemical patterning factors. Higher concentrations of chemical factors in this protocol may result in toxicity (purmorphamine shows beginning toxicity at 1 μM, CHIR99021 at 8 μM and DAPT at 3 μM in this protocol). Different batches of Shh-C24II may have different potencies, thus requiring adjustments in concentration.

### Materials (european catalog numbers)

- Human embryonic stem cells or induced pluripotent stem cells of good quality with minimal spontaneous differentiation. Grown on mouse embryonic feeder cells or in defined medium on matrigel or vitronectin.
- EDTA 0.5 M pH 8.0 (15575–020). Dilute to 0.5 mM in PBS and store at RT.
- DMEM/F-12 (Invitrogen, cat. no. 21331–020).
- Neurobasal (Invitrogen, cat no. 21103–049).
- L-Glutamine (200 mM, Invitrogen, cat. no. 25030–081), aliquot and store at −20°C.
- Accutase (Invitrogen, cat. no. A11105–01), aliquot and store at −20°C.
- B27 supplement without vitamin A (Invitrogen, cat no 12587–010), aliquot and store at −20°C.
- N2 supplement (Invitrogen, cat no 17502–048), aliquot and store at −20°C.
- SB431542 (TGFβ inhibitor, Tocris Bioscience, cat. no. 1614 or Axon Medchem, cat. No. 1661). Make stock aliquots of 20 mM in DMSO and store at −20°C.
- Y-27632 dihydrochloride (ROCK inhibitor; Tocris Bioscience, cat. no. 1254 or Axon Medchem 1683). Make stock aliquots of 10 mM in H_2_O and store at −20°C.
- CHIR99021/CT99021 (GSK3 inhibitor; Axon Medchem, cat no 1386). Make stock aliquots of 10 mM in DMSO and store at −20°C.
- Recombinant human Noggin (R&D Systems, cat. no. 6057-NG)^*^.
- Recombinant human BDNF (R&D Systems, cat. no. 248-BD)^*^.
- Recombinant human GDNF (R&D Systems, cat. no. 212-GD)^*^.
- Recombinant modified human Sonic Hedgehog C24II (R&D Systems; cat. no.1845-SH-025)^*^.
- Purmorphamine (Smoothened agonist; Stemgent 04-0009). Make stock aliquots of 10 mM in DMSO and store at −20°C.
- Ascorbic acid (Sigma, cat. no. A5960). Make stock aliquots of 200 mM in H_2_O and store at −20°C.
- Dibutyryl-cAMP (Sigma cat no. D0627). Make stock aliquots of 50 mM in H_2_O and store at −20°C.
- DAPT (γ-secretase inhibitor; Tocris Bioscience, cat no. 2634). Make stock aliquots of 10 mM in DMSO and store at −20°C.
- Polyornithine (Sigma cat no. P3655). Make sterile filtered stock aliquots of 1.5 mg/ml in H_2_O and store at −20°C.
- Laminin (Invitrogen cat no. 23017-015). Aliquot and store at −80°C.
- Fibronectin (Invitrogen cat no. cat no 33010-018). Make stock aliquots of 0.5 mg/ml in PBS + 5 mM NaOH and store at −20°C.
- Non tissue-culture treated multiwell plastic plates (12- or 24-wells).
- Tissue-culture treated multiwell plastic plates (12-, 24-, or 48-well).
- PBS.
- Sterile water.

^*^For protein growth factors, make stock solutions in PBS + 0.1% BSA and store at −20°C.

### Reagent setup

#### Neural induction medium (NIM):

DMEM/F-12: Neurobasal (1:1)

1× N2 (1:100)
1× B27 (1:50)
2 mM L-Glutamine (1:100)

#### Neural proliferation medium (NPM):

DMEM/F-12: Neurobasal (1:1)

0.5× N2 (1:200)
0.5× B27 (1:100)
2 mM L-Glutamine (1:100)

#### Neural differentiation medium (NDM):

Neurobasal

1× B27 (1:50)
2 mM L-Glutamine (1:100)

## Polyornithin/laminin/fibronectin (PO/lam/FN) coated dishes

For coating, dilute PO stock solutions 1:100 in H_2_O to yield a final concentration of 15 μg/ml. Add the solution to wells and incubate at 37°C for 48 h (i.e., 0.2 ml/cm^2^= 350 ul in a 24-well dish and 700 in a 12-well dish). After 48 h, aspirate the PO solution and wash three times in sterile H_2_O. Prepare FN + lam solution by adding 1:100 of FN (0.5 mg/ml) to PBS in a 50 ml tube. Swirl the tube well to ensure that FN is in solution (ensure that is does not form a precipitate or sticks to the tube wall). Then place the tube at 4°C for 15–30 min to cool down the solution. In the meantime, thaw an aliquot of laminin at 4°C. When the PBS-FN solution is cold, add laminin to the tube to yield a final concentration of 5 μg/ml for each. Swirl the tube. Immediately add the FN/lam solution to the washed PO-coated wells at 0.2 ml/cm^2^ and incubate at 37°C for 48–72 h. Wash the plates twice in PBS prior to plating the cells. Plates incubating with FN/lam solution can be left in the incubator for up to 1 week before use.

## Dilution of CHIR99021/CT99021

Since patterning with CHIR99021 is extremely concentration dependent, it is important that the compound is diluted in exactly the same way for every experiment. The compound should be kept frozen and exposure to light should be minimized to avoid degradation.

- Keep CHIR99021 stock aliquots (10 mM in DMSO) at −20°C.
- These aliquots can be thawed and used up to three times, and in between they should be stored at −20°C.
- For use in experiments, thaw the frozen aliquot and dilute 3 μl in 297 μl medium (1:100) to yield a 100 μM solution. Aim at always using the same pipettes for dilution to avoid drifts in concentration between experiments. Always use freshly prepared dilutions for experiments.
- Mix well and add the 100 μM solution to the cells to yield the desired concentration (i.e., for 0.8 μM final concentration, add 8 μl solution to 1 ml of medium).

### Conflict of interest statement

The authors declare that the research was conducted in the absence of any commercial or financial relationships that could be construed as a potential conflict of interest.

## References

[B1] Chambers S. M. Fasano C. A. Papapetrou E. P. Tomishima M. Sadelain M. Studer L. (2009). Highly efficient neural conversion of human ES and iPS cells by dual inhibition of SMAD signaling. Nat. Biotechnol. 27, 275–280 10.1038/nbt.1529 19252484 PMC2756723

[B2] Fasano C. A. Chambers S. M. Lee G. Tomishima M. J. Studer L. (2010). Efficient derivation of functional floor plate tissue from human embryonic stem cells. Cell Stem Cell 6, 336–347 10.1016/j.stem.2010.03.001 20362538 PMC4336800

[B3] Kirkeby A. Grealish S. Wolf D. A. Nelander J. Wood J. Lundblad M. (2012). Generation of regionally specified neural progenitors and functional neurons from human embryonic stem cells under defined conditions. Cell Rep. 1, 703–714 10.1016/j.celrep.2012.04.009 22813745

[B7] Kirkeby A. Parmar M. (2012). Building authentic midbrain dopaminergic neurons from stem cells—lessons from development. Transl. Neurosci. 3, 314–319

[B8] Kriks S. Shim J. W. Piao J. Ganat Y. M. Wakeman D. R. Xie Z. (2011). Dopamine neurons derived from human ES cells efficiently engraft in animal models of Parkinson's disease. Nature 480, 547–551 10.1038/nature10648 22056989 PMC3245796

[B4] Nat R. Nilbratt M. Narkilahti S. Winblad B. Hovatta O. Nordberg A. (2007). Neurogenic neuroepithelial and radial glial cells generated from six human embryonic stem cell lines in serum-free suspension and adherent cultures. Glia 55, 385–399 10.1002/glia.20463 17152062

[B5] Perrier A. L. Tabar V. Barberi T. Rubio M. E. Bruses J. Topf N. (2004). Derivation of midbrain dopamine neurons from human embryonic stem cells. Proc. Natl. Acad. Sci. U.S.A. 101, 12543–12548 10.1073/pnas.0404700101 15310843 PMC515094

[B6] Zhang S. C. Wernig M. Duncan I. D. Brustle O. Thomson J. A. (2001). *In vitro* differentiation of transplantable neural precursors from human embryonic stem cells. Nat. Biotechnol. 19, 1129–1133 10.1038/nbt1201-1129 11731781

